# Predictive maintenance optimization for industrial equipment via reliable prognosis and risk-aware reinforcement learning

**DOI:** 10.1007/s40747-025-02127-w

**Published:** 2025-11-11

**Authors:** Zifei Xu, Qiang Zhang

**Affiliations:** 1 School of Power and Energy Engineering, University of Shanghai for Science and Technology, Shanghai, China; 2 Institute for Risk and Uncertainty, University of Liverpool, Liverpool, UK

**Keywords:** Predictive maintenance, Prognostics and health management, Uncertainty quantification, Reliability, Risk-aware decision-making

## Abstract

Predictive maintenance (PdM) based on Remaining Useful Life (RUL) prediction plays a crucial role in improving performance and reducing lifecycle costs of industrial equipment. This study proposes an intelligent PdM framework that integrates a RUL prediction model based on probabilistic neural network with a distributional reinforcement learning agent based on QR-DQN. In the first stage, the RUL prediction model is developed to process sensor data to generate accurate RUL predictions, quantify predictive uncertainty, and estimate the probability of failure within a given horizon. Building on the health condition assessment, the QR-DQN agent learns the distribution of long-term maintenance returns and makes sequential decisions among multiple actions. By adopting risk-sensitive decision rules, the agent explicitly accounts for uncertainty and failure risk, achieving a balance between safety, cost efficiency, and timeliness of interventions. Experimental evaluations on complex system degradation demonstrate that the proposed intelligent PdM outperforms conventional baselines by reducing catastrophic failures, optimizing maintenance schedules, and improving overall reliability.

## Introduction

### Background and motivation

Prognostics and Health Management (PHM) in an important prerequisite for determining the effectiveness and optimality of maintenance strategy, including the timing and sequencing of maintenance actions [1], PHM can significantly improve the reliability of the cyber-physics systems(CPS), such as wind turbines, aircraft engines, high-speed rail systems and other critical infrastructure, by providing accurate health assessment and failure prediction during actual lifecycle conditions. Development and implementation of innovative solutions to advance technologies in PHM for predictive maintenance (PdM) help to address the urgent and practical needs of today’s CPS. Accordingly, PdM enhance the safe operation and cost-saving performance of equipment across diverse application domain [2, 3], as it enables the analysis and prediction of system health, and the implementation of preventive actions before failures occur or escalates from minor issues into catastrophic consequences [4–6]. In general, a PdM framework typically consists of three key modules including condition monitoring, health assessment (or prognosis), and decision-making. These modules form a typical information–physical loop in CPS, where the overall reliability and performance of PdM largely depend on the accuracy of health assessment and the effectiveness of maintenance decision-making [7].

### Literature review and research gaps

Rapid advancements in artificial intelligence offers the potential to drive the data-driven-based PdM. The technique has proven to be an effective solution for performance improvement and reducing operation and maintenance (O&M) costs [8–10]: The nature of Real-time state awareness of preventive maintenance and how it updates knowledge regarding maintenance by real-time operational states of engineering systems is another important aspect of PdM. In general, PdM follows a predefined decision-making process, allowing for the implementation of preventive actions, including but not limited to, preventive inspections, maintenance, resource management, and pre-emptive failure analysis and prevention. Furthermore, PdM involves determining an optimal maintenance strategy before the occurrence of events or failures. This strategy fulfils the intelligent and efficient operation and maintenance requirements, resulting in overall performance improvement and cost-saving.

Recent advances in sensing technologies, internet of things (IoTs), and machine learning/deep learning techniques, have provided the impetus for a boom in condition monitoring (CM) based PdM. The CM based PdM consists of remaining useful life (RUL) prediction and optimal maintenance strategy determination [11]. The former forecasts potential failures by analysing monitoring data collected through Condition Monitoring (CM) systems, which reflect the current health conditions of the energy systems [12, 13]. The latter plans maintenance actions based on the predicted RUL with the aim of fully minimizing operational and maintenance costs. Consequently, predictive maintenance represents a promising trend and the future of maintenance strategy planning.

To ensure more reliable and informed operational and maintenance decisions, obtaining accurate and dependable health information from monitoring data is of paramount importance. Regarding prognosis, various approaches have been developed to assess equipment health; among them, Remaining Useful Life (RUL) prediction plays a central role as it provides quantitative and actionable health information. Recently, an increasing number of studies have explored deep learning (DL)–based methods for RUL prediction, which have demonstrated superior capability in capturing nonlinear degradation patterns and handling large-scale sensor data [14]. Majority of these investigations examine critical aspects of RUL prediction models addressing the mentioned task including, robustness and capability enhancement and extensions. The robustness of RUL prediction represents a key and open problem in the sector [15, 16]. To this end, Xiang et al. [17] developed a novel DL network based on a 1-D convolutional neural network (CNN) and multi-head gated recurrent unit to improve the accuracy of the RUL prediction. To improve the robustness of similar tasks, Xiong et al. [18] constructed a physics-informed module aiming at enhancing failure mode classification and improving the robustness of the prognosticator in RUL prediction. Similarly, Yan et al. [19] constructed an RUL prediction model based on the DL technique in which the impact of multi-sensor information fusion on RUL prediction is modelled. It indicates that embedding attention value into a long-short-term memory (LSTM) network contributes to the stability and robustness of RUL prediction. It is noted that using a single DL model in predicting RUL results in less robust estimation due to prediction bias. Therefore, the ensemble models aggregate that use multiple DL models are found to be beneficial because they offer reliable and robust predictions. Thus, Cheng et al. [20] developed a health-state related (HSR) ensemble DL method in consideration of different degradations of the machinery to successfully improve the robustness of the estimated RUL. It is worth mentioning that ensemble DL methods improve the reliability and robustness of predictions which, however, require multiple-data processing and adds burden for model solving and computation.

In terms of DL models’ capability enhancement and extension in RUL predictions, Li et al. [21] integrated domain knowledge into a DL model to improve the reliability of RUL prediction in which a clustering algorithm to transform the original degradation features into embedded vectors and a DL model to accurately predict the RUL are constructed. Additionally, for careful consideration of training/testing data distributions, application of transfer learning has emerged in the RUL prediction as a viable technique. Specifically, Zhang et al. [22] proposed a multistage degradation division method to construct health indicators for RUL prediction in which a transfer learning model is developed based on the multistage health indicators. It is concluded that the predicted RUL offers more accurate models than existing ones.

However, the above studies, rely solely on point RUL estimation which calls for consideration of the uncertainty associated with RUL predictions. Probabilistic neural network has been proposed to equip neural networks with the capability to quantify uncertainty in RUL predictions. For instance, Cao et al. [23] used Monte Carlo (MC) dropout to enable the recurrent neural network to quantify uncertainties in predictions. The proposed framework improves the accuracy of RUL prediction. In addition, Lin et al. [24] developed a Bayesian deep learning (BDL) framework for RUL prediction, which enhances the accuracy of predictions while enabling uncertainty quantification. Additionally, Xu et al. [25] proposed a fully intelligent health indicator as a basis to develop a CNN-LSTM model with a Bayesian neural network for uncertainty analysis of RUL predictions. The above research confirms that consideration of uncertainties contributes to accurate RUL prediction. However, the previous studies failed to consider the impact of uncertainties on the model's reliability.

Although prognosis, particularly RUL prediction, provides valuable insights into the future health state of equipment, its benefits can only be fully realized when combined with decision-making frameworks that translate prognostic outputs into optimal maintenance actions. This has led to extensive research on decision-making strategies, with a growing emphasis on optimization-based and reinforcement learning–driven approaches[26].

Regarding decision-making for maintenance, Zhang et al. [27, 28] considered imperfect repair and accessibility to opportunistic maintenance strategy optimization for O&M cost reduction of wind turbines (WTs). The result indicates that the strategy can significantly reduce O&M costs when compared to traditional preventive maintenance methods. Valet et al. [29] developed an opportunistic maintenance model based on reinforcement learning to increase operational performance, the examination demonstrated that the Reinforcement Learning (RL) policy can deliver an effective combined scheduling strategy by incorporating both internal and external opportunistic possibilities. Besides that, RL has been widely recognized in the domain of safety and reliability. For example, Liu et al. proposed a novel method by integrating RL and system safety method to optimize emergency procedures in dynamic environment [30]. Beyond optimization methods that neglect system degradation, He et al. [31] investigated maintenance optimization using monitored prognostic information for multi-component systems. The results demonstrated that incorporating prognostic information can enhance the robustness of the optimized maintenance strategy. Saleh et al. [32] created a condition-based maintenance framework for maintenance policy optimization to maximize availability and minimize operational costs. Differently, Gonzalo et al. [33] constructed a Genetic Algorithms-Particle Swarm Optimization (GA-PSO) based optimal maintenance strategy for maintenance scheduling of offshore wind turbines with the aim of minimizing cost, which addressed complex optimization problems involving multiple objectives and constraint functions. Lee [34] developed a predictive maintenance framework that utilizes probabilistic convolutional networks and RL, and the results indicate that employing RL for maintenance decisions can effectively reduce overall maintenance costs.

Despite the recent progress in reinforcement learning and prognostics, several critical gaps remain. First, there is a lack of a systematic AI-driven PdM framework that jointly considers both RUL prediction performance and the impact of predictive uncertainty on downstream maintenance decisions. Second, existing studies rarely incorporate more complex maintenance actions such as repairs, with most focusing only on replacements. Third, cost-oriented maintenance decision-making has not been sufficiently investigated in terms of how it may introduce catastrophic risks if predictive uncertainty and safety are ignored. Recent advances in uncertain RL-based optimal control laws [35, 36], and uncertainty-aware learning frameworks [37] provide a solid foundation upon which to build a fully intelligent PdM framework that explicitly accounts for risk, thereby bridging the gap between theoretical reinforcement learning and practical predictive maintenance applications.

### Contributions and innovations

Therefore, this study develops an intelligent PdM framework that integrates a probabilistic neural network–based RUL prognosticator with a distributional reinforcement learning agent based on QR-DQN. The proposed framework advances the state of the art in three aspects. First, the RUL prognosticator not only predicts the expected lifetime but also quantifies uncertainty and estimates failure probability, providing a more reliable health assessment. Second, the decision-making module incorporates imperfect maintenance actions known as repairs in addition to replacements, thereby enabling more realistic and flexible maintenance strategies. Third, by adopting a distributional reinforcement learning approach with risk-sensitive decision rules, the framework explicitly accounts for predictive uncertainty and degradation risk, balancing safety, cost efficiency, and timeliness of interventions. The effectiveness of the proposed framework is demonstrated through experiments on complex system degradation data, where it significantly reduces catastrophic failures, optimizes maintenance schedules, and improves overall reliability compared with conventional baselines.

The structure of the rest of the paper is as follows: “Methodologies” introduces the methodologies for Intelligent PdM. “Experimental setup and evaluation” describes the experimental setup and evaluation. “Results and discussion” presents case studies, and “Conclusions” offers the conclusions.

## Methodologies

**Fig. 1:**
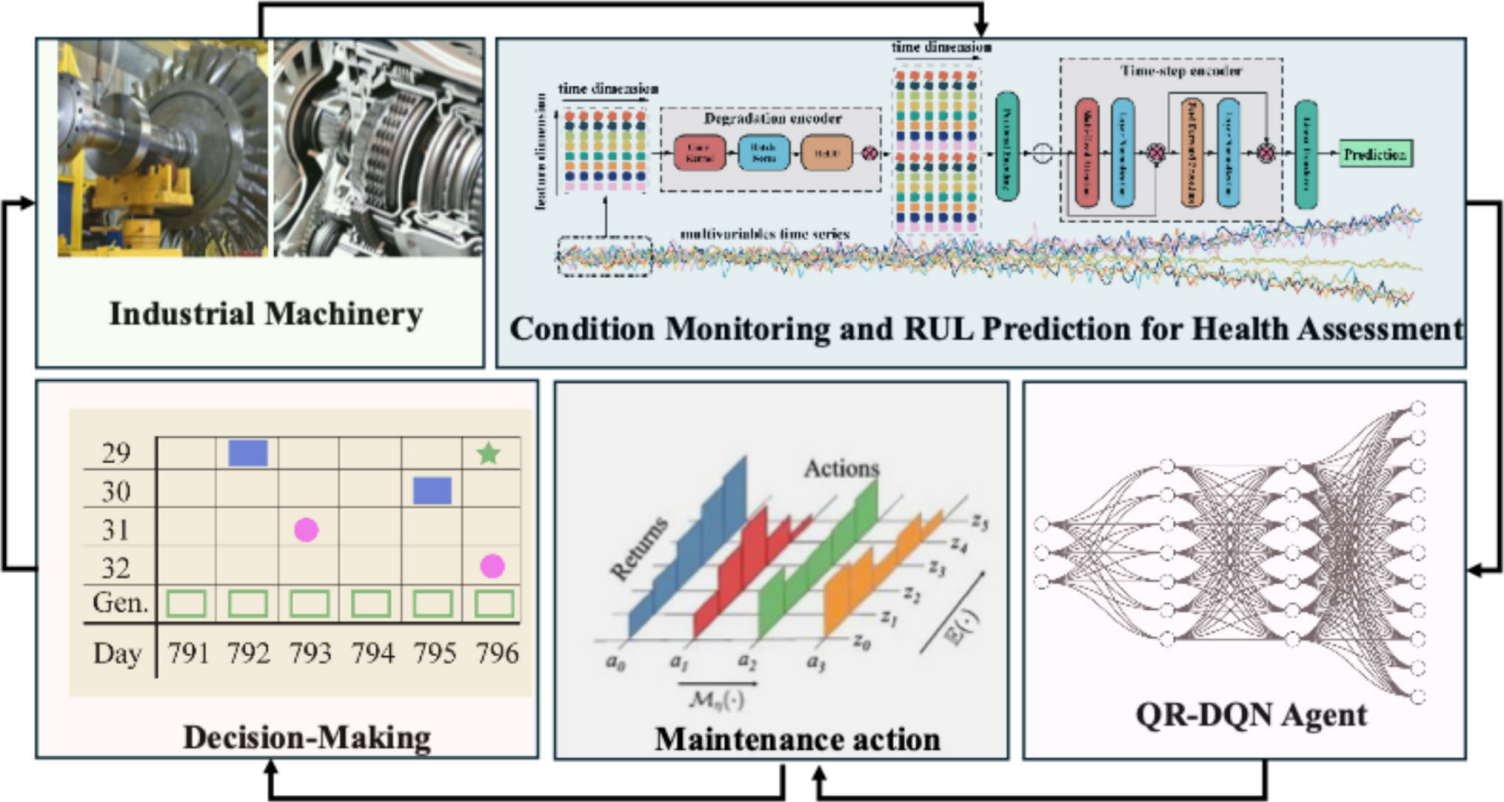
The proposed intelligent PdM framework

In this section, a novel PdM framework is developed based on intelligent RUL prediction and reinforcement learning. In Fig. 1, sensors collect condition data from the target system. Historical data are used to train a RUL predictor offline. The trained model is then embedded into the PdM workflow and coupled with RL agent to select maintenance actions for the CPS. To ensure the reliability of the RUL estimates, Monte Carlo dropout [38] procedure is used to quantify predictive uncertainty. The RL agent receives both the average RUL and its associated uncertainty as observations. The action space comprises three maintenance options, and the negative of each action’s cost is used as the reward for policy optimization. Overall, the PdM framework is reliable and robust, provided that RUL predictions are trustworthy and the maintenance policy explicitly accounts for decision risk through uncertainty-aware reasoning.

Figure 1, this framework illustrates an intelligent predictive maintenance (PdM) system. Industrial machinery generates operational data through sensors, which are processed in the condition monitoring and RUL prediction module to provide a quantitative health assessment. The health state is then fed into a QR-DQN agent, which employs distributional reinforcement learning to model the long-term returns of different maintenance strategies. Based on this, the agent produces a probability distribution over possible maintenance actions, enabling optimization of decisions under uncertainty. Finally, the decision-making module integrates system state, risk, and cost considerations to derive an effective maintenance policy, forming a closed-loop PdM process for reliable and intelligent management of complex industrial assets

### RUL prediction

**Fig. 2:**
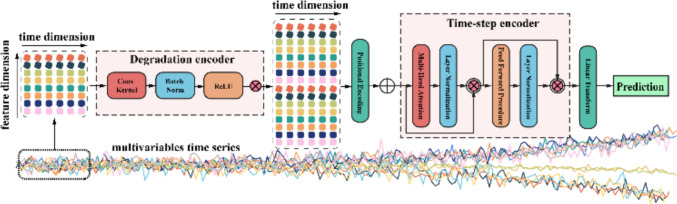
RUL prognosticator framework

The traditional neural network is unable to quantify the prediction's uncertainty as it often leads to overconfident RUL prediction. To overcome the limitation of the point-estimated predictor, in this study, the probabilistic prognosticator is a combination of self-attention, convolutional neural network and. Specifically, the convolutional module enables the RUL predictor to extract useful information from multivariable degradation features. Self-attention module enables the prognosticator to have the ability to capture the time-dependent relationship between the degradation features to predict the RUL of the next state based on the current state. The Monte Carlo dropout. Figure 2 presents the schematic view of the proposed RUL predictor, which uses degradation features to predict the RUL of engineering systems.

In this study, multiple degradation signals collected from different sensors are used as the input features for RUL predictor training. To effectively handle the anisotropic behaviours of these multivariate degradation features, a convolutional neural network module, called degradation features encoder, is applied to each observation point in chronological order to reduce the dimension of the multivariable degradation features. This encoder serves as an embedding process that transforms heterogeneous sensor signals at each observation point into a compact and informative representation in chronological order. The embedded features are then fed into a self-attention module for temporal sequence encoding, followed by a fully connected layer to estimate the RUL. To capture predictive uncertainty, dropout is applied to all neural network units involving weight computation. By combining the strengths of convolutional neural networks for feature embedding and self-attention networks for temporal modelling.

#### Degradation encoder

To extract representative degradation features from multiple monitored signals for the downstream RUL prediction task, we consider one-dimensional multivariate degradation signal ${\mathbb{X}}_{t}^{{n}_{ }}\in {\mathfrak{R}}^{n\times \mathrm{T}}(t=1,2,3\dots , T)$ with $n$-dimensional variables. At each time step, the convolution kernel scans across these multivariate inputs, effectively mapping the raw degradation features into a latent representation. This process can be regarded as an embedding of the degradation features, where the subsequent activation and regularization functions, defined in Eq. (1), are applied to introduce nonlinearity and stabilize the feature distribution.

1 $${\mathbb{X}}_{t}^{k} =\mathit{norm}\{\sigma (\mathcal{W}*{\mathbb{X}}_{t}^{{n}_{ }})\}$$

In the input matrix of the degradation signal ${\mathbb{X}}_{t}^{{n}_{ }}$, $\mathcal{W}$ is the weight matrix of the convolutional kernel, $\sigma \left( \cdot \right)$ represents an activation function and $norm\{ \cdot \}$ denotes a normalization function. Due to multi-layer convolution procedure, the variable size ${{\boldsymbol{X}}}_{t}^{{n}_{ }}$ would be changed from $n$ to $k$-dimentional or filtered features ${{\boldsymbol{X}}}_{t}^{k}$, where ${{\boldsymbol{X}}}_{t}^{k}\in {\mathfrak{R}}^{k}$. The filter features will be the embedding features for time-step encoder learning.

#### Time-step encoder

Based on the extracted embedding features, the next task is to connect the degradation features with RUL labels step by step. The self-attention mechanism has been widely used in natural language processing. Compared to RNNs, such as LSTM, self-attention network can capture global dependencies among the embedding features at each degradation point, which lead to better overall understanding and prediction of RUL.

As shown in Fig. 2, the input ${{\boldsymbol{X}}}_{t}^{k}$ with $k$ -dimensional embedding features is proceed through positional encoding, multi-head attention module, feed-forward procedure and layer normalization for time-step encoding [39]. Equations (2) and (3) encode the time position with $k$-dimensional features.

2 $$P\left(2k\right)=\mathrm{sin}(t/{max\_lengths}^{\frac{2k}{{d}_{model}}})$$

3 $$P\left(2k+1\right)=\mathrm{cos}(t/{max\_length}^{\frac{2k}{{d}_{model}}})$$

where $t$ is the position that needs to be encoding. $k$ is the feature dimension, which leads to self-attention can record the time position relationship. Due to the features ${{\boldsymbol{X}}}_{t}^{k}\in {\mathfrak{R}}^{k\times \mathrm{T}}(t= 1,2,3\dots , T)$ has been proceed by degradation encoder, ${d}_{model}=k$ in this research, which is embedded by linear transform with three independent learnable parameters ${\mathcal{W}}_{\boldsymbol{ }}\boldsymbol{ }\in {\mathfrak{R}}^{{d}_{model}\times k}$ to generate Queries (Q), Keys (K), Values (V). The weighted feature by self-attention is calculated by Eq. (4).

4 $${{\boldsymbol{Z}}}_{t} =softmax(\frac{{Q}_{t}{{K}_{t}{\prime}}}{\sqrt{k}})\cdot {{\boldsymbol{V}}}_{t}$$

where the parameters of ${Q}_{t}$ is ${\mathcal{W}}_{Q}\in {\mathfrak{R}}^{{d}_{model}\times 1}$ for single layer attention. If used multi-head attention, the final weighted features will consist of $NumHeads$ weighted features by Eq. (5)

5 $${\mathrm{head}}_{NumHeads}={Z}_{t}^{NumHeads}$$

Where the projections are parameter for each head is ${\mathcal{W}}_{Q}\in {\mathfrak{R}}^{{d}_{model}\times {d}_{k}}$ to the ${Q}_{t}$. ${d}_{k}={d}_{model}/NumHeads$, same to the ${K}_{t}$ and ${V}_{t}$. The resulting weighted features are then passed through residual connections and layer normalization. Finally, a multilayer perception maps the high-level embeddings into RUL predictions via regression.

Algorithm 1 summarizes the end-to-end workflow of model optimization.

**Algorithm 1:**
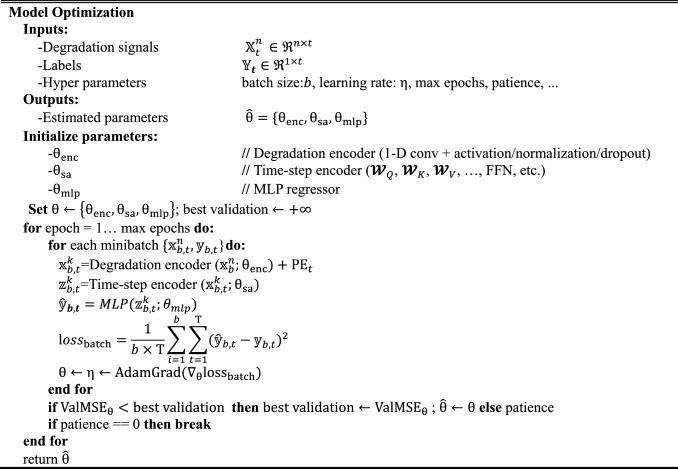
Training of Conv SA RUL Predictor

#### Uncertainty quantification

In this study, the RUL prediction is defined as:

6 $${Y}=f({\mathbb{X}}_{t}|\theta )$$

where ${\mathbb{X}}_{t}\in {\mathfrak{R}}^{n}(t= 1,2,3\dots , T)$ denotes the monitored data at time step $t$ with $n$-dimensional features. The RUL predictor $f$ is instantiated by the proposed Conv-SA network. To further account for uncertainty in RUL prediction, the deterministic mapping in Eq. (7) is extended into a probabilistic form:

7 $${p(Y|X,\theta )}=f({\mathbb{X}}_{t}|\theta )$$

where dropout-based parameter sampling provides a practical way to approximate epistemic uncertainty in predictions. Specifically, given an input ${\mathbb{X}}_{t}$, we perform $N$ stochastic forward passes through the Conv–SA prediction model with dropout activated, yielding a set of RUL estimates ${\{{\widehat{\mathrm{RUL}}}_{t}^{(i)}\}}_{i=1}^{N}$. The predictive mean is used as the final RUL estimate ${\overline{\mathrm{RUL}} }_{t}=\frac{1}{N}\sum_{i=1}^{N}{\widehat{\mathrm{RUL}}}_{t}^{(i)}$, while the variance reflects the epistemic uncertainty ${\sigma }^{2}=\frac{1}{N}\sum_{i=1}^{N}{{(\widehat{\mathrm{RUL}}}_{t}^{(i)}-{\overline{\mathrm{RUL}} }_{t})}^{2}$ of the model. Based on this ensemble, cumulative failure probability within $\tau$ days by the empirical CDF of the RUL samples:

8 $${{\widehat{\mathrm{CFP}}}_{t}\left(\tau \right)=}{\rm P}({RUL}_{t}\le \tau |{\mathbb{X}}_{t})$$

where ${RUL}_{\mathrm{t}}$ denotes the RUL random variable at time $t$ obtained from multiple stochastic inference of the RUL predictor; ${\rm P}( \cdot )$ is the probability that the asset will fail within $\tau$ days given the current observations ${\mathbb{X}}_{t}$. Accordingly, average RUL estimation, epistemic uncertainty and empirical CFP are used as the state in RL.

### Predictive maintenance framework

In this section, reinforcement learning is used as a basic framework to develop a novel approach for PdM of the cyber-physical systems. A central challenge is to specify an action space and a reward function that faithfully emulate real maintenance activities, most notably repair and replacement, so that the learned policy is operationally meaningful. We embedded the Conv-SA RUL predictor (“RUL prediction”) directly into the RL environment. At each decision epoch, the environment queries the predictor to obtain the current RUL estimate and treats it as the health state fed to the agent. The agent then chooses one of three maintenance actions: do nothing (defer to the next inspection horizon), repair (partial life restoration with downtime), or replace (full restoration at higher cost). The immediate reward encodes the economic consequence of that choice. It subtracts the direct maintenance cost and adds penalties when decisions are too late (leading to failure) or too early (wasting RUL). When a repair meaningfully extends usable life, the reward reflects that benefit; otherwise only a small service cost is charged. Episodes terminate at failure or planned replacement.

#### Maintenance decision-making optimization based on quantile regression deep Q network

DQN and their improved variants such as Dual-DQN are widely used RL approaches for discrete decision-making problems. However, these approaches mainly optimize the expected reward and therefore fail to explicitly account for the risks caused by suboptimal decisions. This limitation becomes critical in industrial O&M scenarios, where inappropriate decisions may lead to catastrophic consequences, particularly for critical infrastructures or other equipment. To address this issue, this section develops an end-to-end PdM decision-making framework based on QR-DQN. By integrating the outputs of a RUL prediction module, the proposed approach enables risk-aware decision optimization, which not only improves reliability but also reduces the probability of high-impact failures. Algorithm 2 provides the proposed Risk-aware QR-DQN for PdM.

**Algorithm 2:**
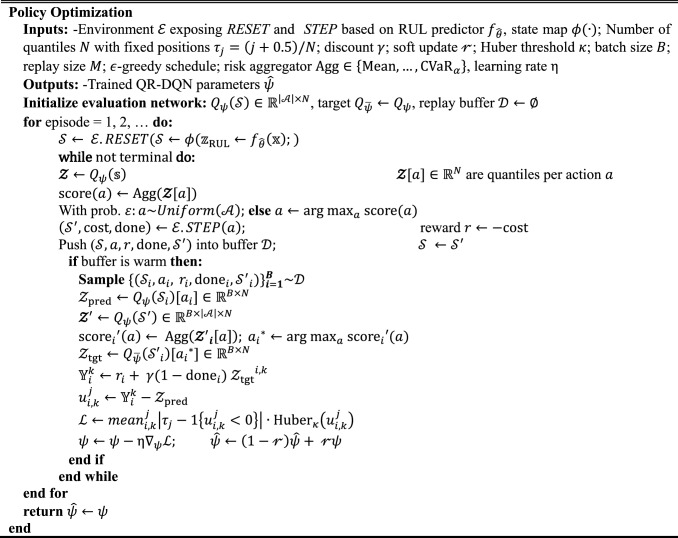
Risk aware QR DQN for PdM (Training)

This algorithm builds a risk-aware PdM controller by combining QR-DQN with the Conv-SA RUL predictor from “RUL prediction”. At decision epoch $t$, the environment runs ${f}_{\widehat{\theta }}$ on the current sensor ${\mathbb{X}}_{t}$ and return the state ${\mathcal{S}}_{t}$.

9 $${\mathcal{S}}_{t}=[{\overline{\mathrm{RUL}} }_{t},{{{{\sigma }_{t}}^{2},\widehat{\mathrm{CFP}}}_{t}\left(\tau \right)}]$$

Given ${\mathcal{S}}_{t}$ the agent uses QR-DQN to compute distributional action-values ${\mathcal{Z}\leftarrow Q}_{\psi }\left(\mathcal{S}\right)\in {\mathbb{R}}^{\left|\mathcal{A}\right|\times N}$ and and converts them into scores via a risk aggregator score(a) ← Agg(Z[a]), where $\mathrm{Agg}\in \left\{\mathrm{Mean}, \dots ,{\mathrm{CVaR}}_{\alpha }\right\}$ that can control the risk of the decision. Then the agent selects an action of maintenance ${\alpha }_{t}\in \{0: \;\text{Do Noting},1:\;\mathrm{repair},2:\;\mathrm{replacement}\}$. The definitions of repair, replacement the cost associated with each action are specified in “Maintenance actions and reward formulation”. The immediate reward follows the cost-minization conventions ${r}_{t}=R\left({s}_{t},{\alpha }_{t}\right)=-(C\left({\alpha }_{t}\right),P\left({\alpha }_{t},{\widehat{\mathrm{RUL}}}_{t},\mathrm{RUL}\right))$, where $C\left({\alpha }_{t}\right)$ is the basic maintenance cost, and $P( \cdot )$ denotes penalties for failure or wasted lifetime, which couples the predictive signal to economic outcomes. This closed-loop integration allows the agent to leverage data-driven RUL predictions to optimize flexible operation-and-maintenance decisions, yielding an end-to-end intelligent PdM framework.

#### Maintenance actions and reward formulation

This section demonstrates the way of achieving predictive maintenance based on probabilistic RUL estimation in consideration of imperfect and perfect maintenance operations (repair and replacement). This approach is more aligned with industry practices compared to existing research because engineering systems typically undergo repair throughout their lifecycle rather than being replaced.

**Fig. 3:**
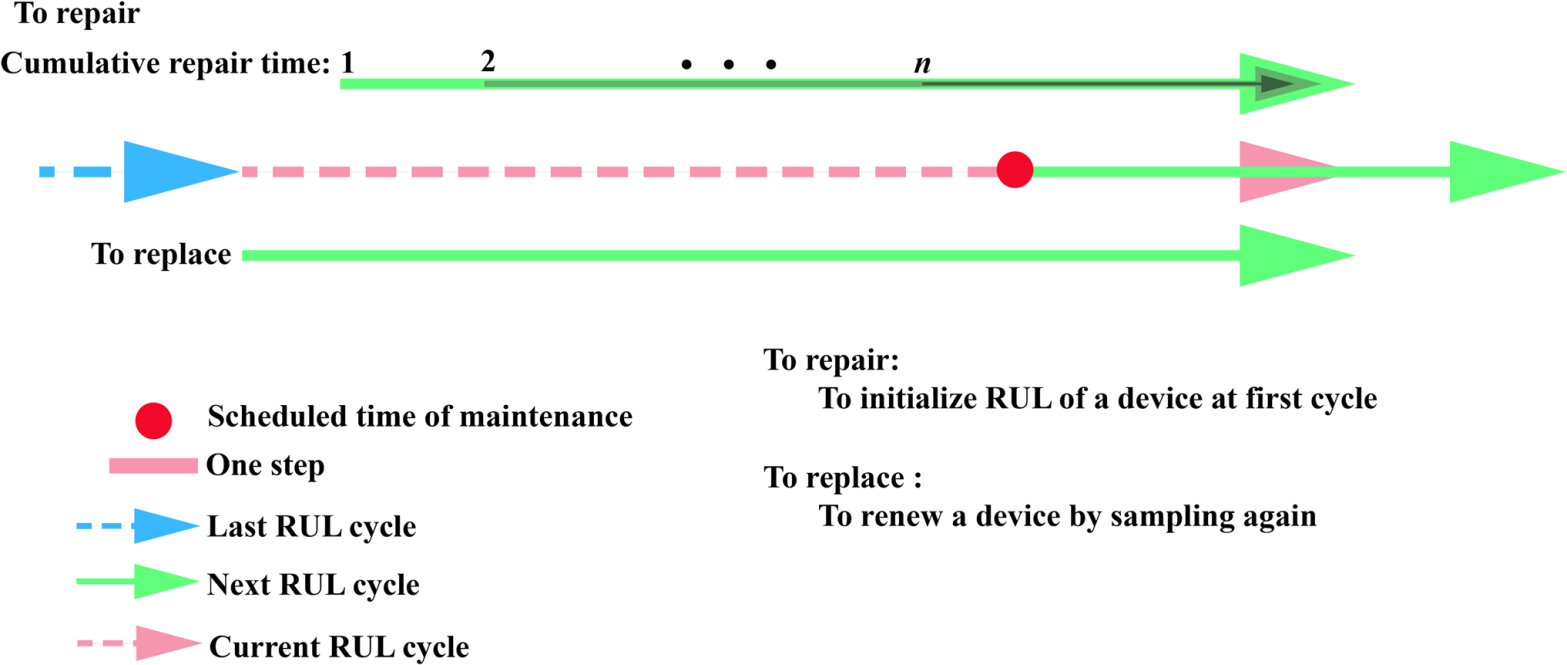
Maintenance planning at one decision cycle

Figure 3 illustrates the maintenance strategy adopted, which involves two primary operations: repair and replacement. These maintenance operations are scheduled at predetermined maintenance points. In the case of component replacement, a new device is selected from the sample pool. If component repair is performed, the initial sampled point of the RUL is reset. However, it is important to note that continuous repairs on the same component do not maintain a stable and healthy RUL state. Therefore, if consecutive repairs are carried out on the same device, the cumulative number of repairs is tracked, and a penalty is imposed on the initial RUL state based on the number of repairs. As depicted in Fig. 3, performing the repair operation multiple times results in a significantly reduced RUL.

At each decision epoch $t$, let ${\overline{\mathrm{RUL}} }_{t}$ denote the predicted mean predicted RUL (Eq. (6)) from the embedded Conv-SA model, ${{\sigma }_{t}}^{2}$ is the predictive uncertainty from Eq. (7), and ${\widehat{\mathrm{CFP}}}_{t}\left(\tau \right)$ is the conditional failure probability following Eq. (8). The environment maintains a device-specific RUL cap ${\mathrm{R}}_{max}$ inferred from the asset with a global fallback. Each action ${\alpha }_{t}\in \{0:\;\text{Do Noting},1:\;\mathrm{repair},2:\;\mathrm{replacement}\}$ with a scheduled delay $n\in N$ days. The details of each action for maintenance and corresponding cost are defined as follows:

At each decision epoch $t$, the immediate reward is defined under the cost-minimization convention as ${r}_{t}=-C\left({a}_{t};{\mathcal{S}}_{t},\mathrm{history}\right)$, where a terminal failure yields a fixed penalty ${r}_{t}={C}_{fail}<0$. Three types of actions are considered: do nothing, repair, and replacement, each associated with a specific cost formulation.

For the do-nothing action (${a}_{t}=0$), the system continues its operation over a fixed cycle length ${t}_{0}(\mathrm{e}.\mathrm{g}., 30 \;\mathrm{days}).$ The corresponding cost includes both a holding term and a risk term determined by the conditional failure within ${t}_{0}$, expressed as

10 $${C}_{0}={\lambda }_{\mathrm{hold}}{t}_{0}+{\lambda }_{\mathrm{risk}}{\widehat{\mathrm{CFP}}}_{t}\left({t}_{0}\right)$$

When performing repair (${a}_{t}=1$), the action is scheduled with a delay $t=\mathrm{max}\{1,\mathrm{action}\_\mathrm{day}\}$, and tis cost depends on the repair expense, downtime, risk exposure, prior repair history, and the effective restoration of the RUL. Specially, the cost is given by

11 $$\begin{aligned}{C}_{1}={{c}_{\mathrm{rep}}+c}_{\mathrm{down}}s+{\lambda }_{\mathrm{risk}}{\widehat{\mathrm{CFP}}}_{t}\left(s\right)+{{\omega }_{\mathrm{surch}}(n+1)}^{{p}_{\mathrm{surch}}}-{\omega }_{\Delta \mathrm{RUL}}\times \Delta \mathrm{RUL}\end{aligned}$$

where $\Delta \mathrm{RUL}$ is the restored life is credited after repair, The restoration is computed from:

12 $${\mathrm{RUL}}^{\mathrm{target}}=\mathrm{min}\left\{{\mathrm{RUL}}^{\mathrm{before}}+\alpha \left(n\right){\bullet \mathrm{RUL}}^{\mathrm{before}},{\mathrm{R}}_{\mathrm{max}}\right\}$$

where ${\mathrm{RUL}}^{\mathrm{before}}=\mathrm{max}\left\{0,{\widehat{\mathrm{RUL}}}_{t}-t\right\}$; $\alpha \left(n\right)$ denotes the repair efficacy factor after $n$ prior repairs., modeled as an exponentially decaying function ${\alpha }_{0}{e}^{-\beta n}$ bounded within [${\alpha }_{\mathrm{min}},{\alpha }_{\mathrm{max}}$]. This formulation captures the practical observation that the effectiveness of successive repairs diminishes over time: initial repairs can restore a larger portion of the remaining life, whereas later repairs provide limited benefit. Thus, the effective restoration is:

13 $$\Delta \mathrm{RUL}=\mathrm{max}\left\{0,{\mathrm{RUL}}^{\mathrm{target}}-{\mathrm{RUL}}^{\mathrm{before}}\right\}$$

For the replacement action (${a}_{t}=2$), in addition to the delay replacement, the associated cost accounts for both replacement expense and wasted lifetime prior to replacement, formulated as

14 $${C}_{2}={c}_{\mathrm{repl}}{+\lambda }_{\mathrm{waste}}\times \mathrm{waste}$$

where the wasted life is given by $\mathrm{waste}=\mathrm{max}\{0,{\widehat{\mathrm{RUL}}}_{t}-t\}$. After replacement, the system resumes from a renewed unit with RUL close to ${R}_{\mathrm{max}}$.

In all non-terminal transitions, the reward is unified as ${r}_{t}=-{C}_{{a}_{t}}$ with ${C}_{{a}_{t}}\in \{{C}_{0},{C}_{1},{C}_{2}\}$. The complete set of hyperparameters governing these formulations is summarized in Table 1, which were determined by combining empirical practice in the PdM literature with normalization considerations for our experimental setting. Specifically, the relative magnitudes of replacement cost, repair cost, and wasted-life penalty were chosen in line with classical cost models for preventive and corrective maintenance [40]. The weighting on conditional failure probability and the terminal failure penalty were calibrated to reflect the disproportionate impact of catastrophic failures compared with routine interventions, following the risk-sensitive maintenance optimization framework [41]. Moreover, the repair efficacy factor was modeled as an exponentially decaying function of the number of prior repairs, bounded within practical limits, consistent with the imperfect maintenance literature [42, 43]. While the absolute numerical values are normalized for simulation convenience, their relative scales capture realistic trade-offs between downtime, cost, and reliability as supported by prior studies.

**Table 1:** Hyperparameters governing PdM formulations

Symbol	Meaning	Value used in this study
${\mathrm{R}}_{\mathrm{max}}$	Device-specific RUL cap	150 (default, inferred per asset)
${t}_{0}$	Do-nothing cycle (days)	30
${\lambda }_{\mathrm{risk}}$	Weight on CFP risk	6.0
${\lambda }_{\mathrm{hold}}$	Holding/idle cost per day	0.02
${c}_{\mathrm{repl}}$	Base replacement cost	6.0
${\lambda }_{\mathrm{waste}}$	Wasted-life penalty weight	0.015
${c}_{\mathrm{rep}}$	Base repair cost	3.0
${c}_{\mathrm{down}}$	Repair downtime cost per day	0.02
${\omega }_{\mathrm{surch}}$	Cumulative repair surcharge weight	0.5
${p}_{\mathrm{surch}}$	Surcharge exponent	1.5
${\omega }_{\Delta \mathrm{RUL}}$	Credit per restored RUL	0.005
${\alpha }_{0},\beta$	Repair efficacy base/decay	1.0/0.35
${\alpha }_{\mathrm{min}},{\alpha }_{\mathrm{max}}$	Efficacy bounds	0.10/1.00
${C}_{\mathrm{fail}}$	Terminal failure penalty	− 80

The best performing result for each metric is highlighted in bold

## Experimental setup and evaluation

### Dataset description and hyper-parameters setups

The C-MAPSS dataset, developed by NASA, is widely recognized as a benchmark for research on prognostics and health management (PHM) of complex systems. It provides a series of multivariate time-series datasets that represent the performance degradation of turbofan aircraft engines, a typical cyber-physical system where sensor-based condition monitoring plays a crucial role. In this dataset, degradation trajectories are generated under varying operating conditions and fault modes, with features extracted from multiple sensor channels. This makes C-MAPSS particularly suitable for validating the effectiveness and reliability of the proposed methods in real-world maintenance decision-making contexts.

In this study, four subsets of the dataset (FD001–FD004) are employed to demonstrate both the reliability of the RUL prediction model and the predictive maintenance decision-making framework. Within the maintenance decision-making scenario, perfect maintenance (replacement) is defined as retrieving a new engine from the pool and resetting it to its initial healthy state, whereas imperfect maintenance (repair) is modeled by restoring the RUL of the same engine without replacement, thereby simulating realistic imperfect maintenance conditions.

All experiments are conducted on a Windows 11 operating system with 64 GB of RAM, an Intel Core CPU i9-12900 K, and an RTX A5500 GPU. The neural network model is developed using PyTorch. The prognosticator consists of convolutional neural networks (CNNs) and self-attention networks. A dropout rate of 50% is applied between each neural layer. The size of the convolution kernel is 3, with no padding procedure. There are three convolution kernels in the degradation encoder, with the number of channels increasing based on the number of input features to 64.

For the self-attention network, there are four heads in the self-attention modules, and a dropout rate of 10% is applied between the attention and forward layers. The mini-batch size for training the degradation encoder is 20, and the optimizer used is the Adam optimizer with an initial learning rate of 0.01.

Regarding the decision-making framework training, the initial learning rate is set at 0.001. The target network update frequency is 100, the replay buffer size is 104, and the batch size is 4096.

### Evaluation protocol and metrics for maintenance

To validate the effectiveness of the proposed method, we compare it with four maintenance strategies: (i) DQN-RO (Replace Only baseline): A reinforcement learning scheme that schedules replacements only, without considering repairs.; (ii) An extended baseline that incorporates both repair and replacement within the DQN framework.; (iii) Risk-Neutral PdM: A predictive maintenance policy built on QR-DQN, optimizing expected returns across repair and replacement actions without explicit risk sensitivity.; (iv) Risk-Averse PdM (Proposed): Our final method, which leverages distributional reinforcement learning with QR-DQN. Actions (do nothing, repair, replace) are selected using risk-averse decision rules, yielding a conservative policy that balances failure risk, cost, and time constraints, while reward shaping prevents excessive repairs.

The proposed PdM approach and other methods are evaluated though MC simulation over a fixed duration ${T}_{\mathrm{sim}}$. Within each episode, the agent sequentially selects actions until the end of the simulation horizon, or a terminal failure occurs. During each simulation run, we record maintenance actions. For episode $i$, let ${n}_{i,1}$ and ${n}_{i,2}$ denote the number of repair and replacement actions, respectively, within ${T}_{\mathrm{sim}}$. The action composition is expressed as:

15 $${P}_{a}=\frac{1}{N}\sum_{i=1}^{N}\frac{{n}_{i,1}}{{n}_{i,0}+{n}_{i,1}+{n}_{i,2}},a\in \{0, \mathrm{1,2}\}$$

where $N$ is the total number of simulation episodes ${T}_{\mathrm{sim}}$ and $a=\mathrm{0,1},2$ correspond to do nothing, repair, and replace.

A catastrophe is defined as an episode ending in system failure before ${T}_{\mathrm{sim}}$. Let the indicator

16 $${\mathbb{l}}_{i}^{\mathrm{cat}}=\left\{\begin{array}{c}1, \; if  \;failure \; occurs \; in  \;episode i\\ 0,  \; otherwise,\end{array}\right.$$

then the catastrophe rate is calculated as $CatRate=\frac{1}{N}\sum_{i=1}^{N}{\mathbb{l}}_{i}^{\mathrm{cat}}$.

The Time-To-Replacement (TTR) is proposed as an evaluation metric inspired by the classical reliability index Mean Time to Failure (MTTF). While MTTF measures the expected lifetime until failure under no maintenance, TTR reflects the expected time until the first replacement under a given maintenance policy, thus characterizing the timeliness of preventive interventions. Let ${{c}_{\mathrm{rep}}}^{i}$ denote the first decision epoch when a replacement $a$ = 2 is taken in episode $i$. The TTR is defined as

17 $${\mathrm{TTR}}_{i}=\left\{\begin{array}{c}{{c}_{\mathrm{rep}}}^{i}, if failure occurs within {[0,T}_{\mathrm{sim}}]\\ \varnothing , otherwise,\end{array}\right.$$

## Results and siscussion

### Prognostic performance analysis and comparisons

The reliability and accuracy of predicted RUL play a critical role in maintenance decision-making. To evaluate the effectiveness of the proposed RUL predictor, its performance was validated on aero-engine datasets and compared against several state-of-the-art RUL prediction approaches including GCN [44], SAN [45], LSTM [46], Dual-SA [47] and CNN [48].

**Fig. 4:**
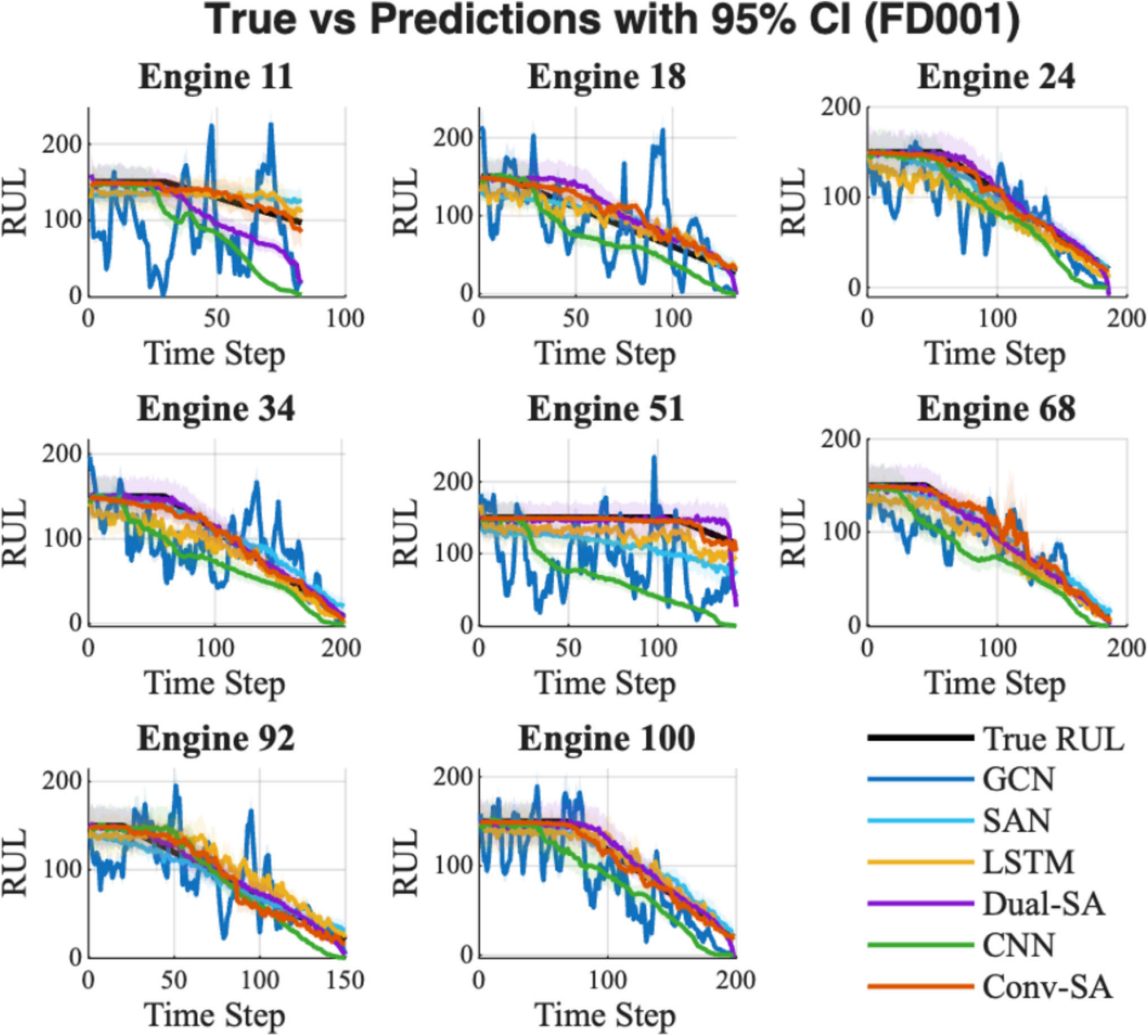
RUL prediction with 95% confidence interval for FD001

Table 2 reports the statistical results of each model for RUL prediction on FD001, while Fig. 4 depicts the lifecycle predictions with the corresponding 95% confidence interval.

**Table 2:** Reliability and superiority evaluation of RUL prediction under FD001

Method name	RMSE $(\overline{x }\pm \sigma )$	Epistemic uncertainty	p-value vs Conv-SA
Conv-SA	$15.82\pm 10.34$	**3.60**	–
GCN	$57.55 \pm 18.02$	4.99	$1.07\times {10}^{-38}$
SAN	$20.30\pm 11.79$	6.35	$4.17\times {10}^{-4}$
LSTM	$21.10\pm 9.88$	9.05	$1.64\times {10}^{-5}$
Dual-SA	$18.89\pm 13.34$	10.06	$3.21\times {10}^{-2}$
CNN	$56.04\pm 22.35$	6.38	$6.83\times {10}^{-32}$

The best performing result for each metric is highlighted in bold

In Table 2, the proposed method Conv-SA achieves the lowest RMSE, clearly outperforming all baseline models. Compared with other advanced approaches, Conv-SA demonstrates a distinct advantage in both mean accuracy and prediction stability. With respect to epistemic uncertainty, Conv-SA attains the lowest value, indicating higher confidence and reliability in its predictions. In contrast, LSTM and Dual-SA exhibit larger uncertainties, implying less stable performance, although LSTM maintains relatively consistent results across the full testing dataset. A lower epistemic uncertainty implies that the predicted RUL is more trustworthy, which is particularly beneficial for maintenance decision-making. Moreover, all comparisons yield extremely small p-values, confirming that the superiority of Conv-SA is statistically significant. In Fig. 4, the predicted curves of Conv-SA (red line) consistently track the ground truth with minimal deviation. The confidence intervals are also narrower compared to those of other models, indicating more reliable predictions, which is consistent with the epistemic uncertainty statistics reported in Table 1. Conv-SA effectively captures the degradation trend and aligns well with the actual failure progression, whereas other models exhibit oscillations or premature convergence, resulting in less accurate lifecycle predictions.

**Fig. 5:**
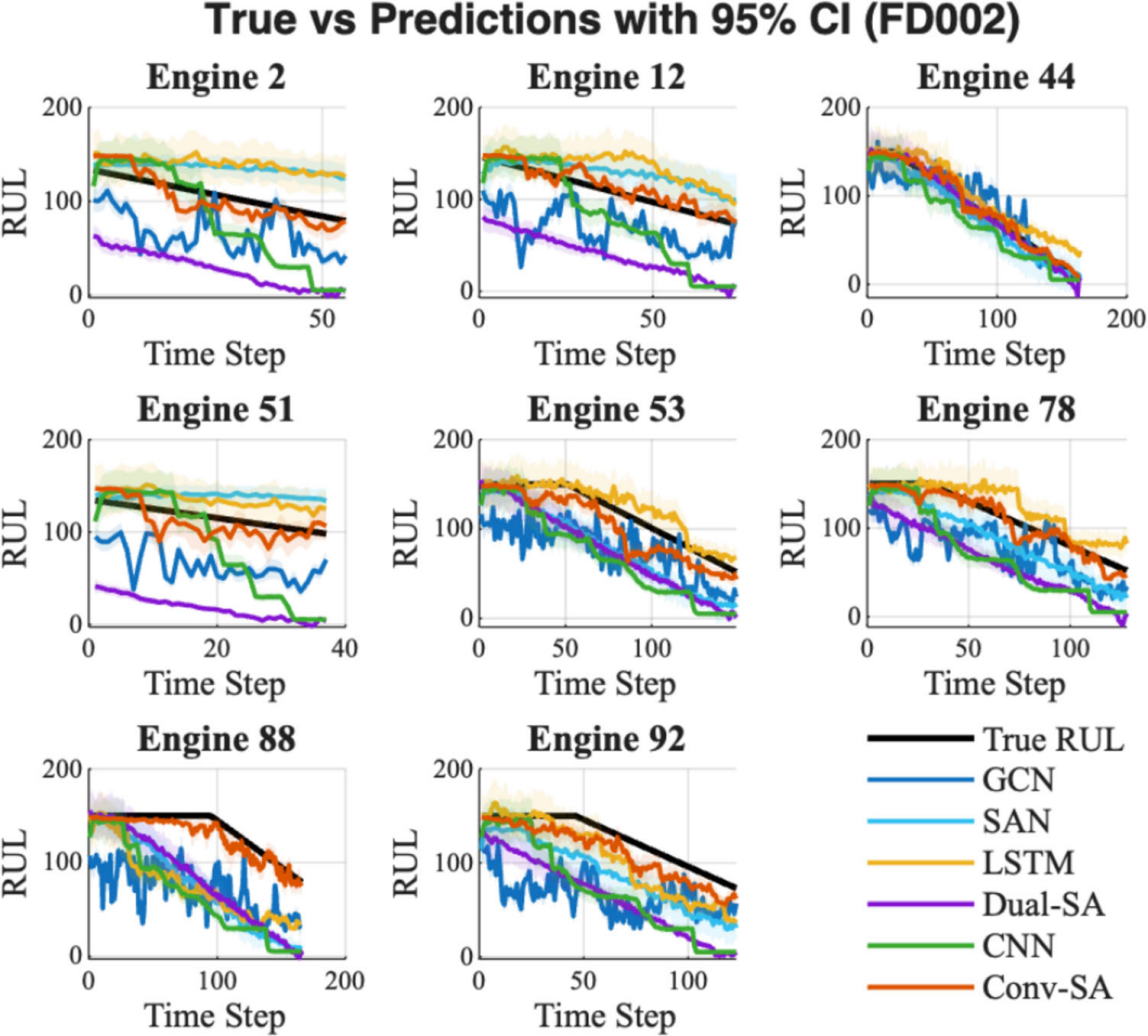
RUL prediction with 95% confidence interval for FD002

Table 2 reports the statistical results of each model for RUL prediction on FD002, while Fig. 5 depicts the lifecycle predictions with the corresponding 95% confidence interval. In Table 2, the proposed method Conv-SA again achieves the lowest RMSE (**25.61 ± 12.84**), clearly outperforming all baseline models. Compared with other advanced approaches, Conv-SA demonstrates superior mean accuracy and stability. In terms of epistemic uncertainty, Conv-SA records the lowest value (**4.38**), highlighting its higher confidence and reliability in prediction. By contrast, LSTM and SAN exhibit larger uncertainties (9.33 and 8.02, respectively), implying less stable performance, even though LSTM achieves a relatively competitive RMSE. A lower epistemic uncertainty means that the predicted RUL is more trustworthy, which is particularly advantageous for maintenance decision-making. Moreover, almost all comparisons yield extremely small p-values, further confirming the statistical significance of Conv-SA’s superiority.

In Fig. 5, Conv-SA’s predicted curves (red line) consistently track the ground truth with minimal deviation across different engines. The confidence intervals for Conv-SA are narrower compared to those of other models, indicating more reliable and stable predictions, which aligns well with the epistemic uncertainty statistics in Table 2. Conv-SA effectively captures the degradation trajectory and matches the actual failure progression, whereas other models (e.g., CNN, GCN, and Dual-SA) often show greater deviations, oscillations, or premature convergence, leading to less accurate lifecycle predictions (Table 3).

**Table 3:** Reliability and superiority evaluation of RUL prediction under FD002

Method name	RMSE $(\overline{x }\pm \sigma )$	Epistemic uncertainty	p-value vs Conv-SA
Conv-SA	$25.61\pm 12.84$	**4.38**	–
GCN	$58.42 \pm 22.04$	4.62	$2.11\times {10}^{-84}$
SAN	$31.66 \pm 21.19$	8.02	$1.97\times {10}^{-6}$
LSTM	$26.83 \pm 18.75$	9.33	$0.316$
Dual-SA	$65.49 \pm 41.13$	5.15	$2.31\times {10}^{-44}$
CNN	$58.65 \pm 24.63$	6.18	$3.83\times {10}^{-76}$

The best performing result for each metric is highlighted in bold

**Fig. 6:**
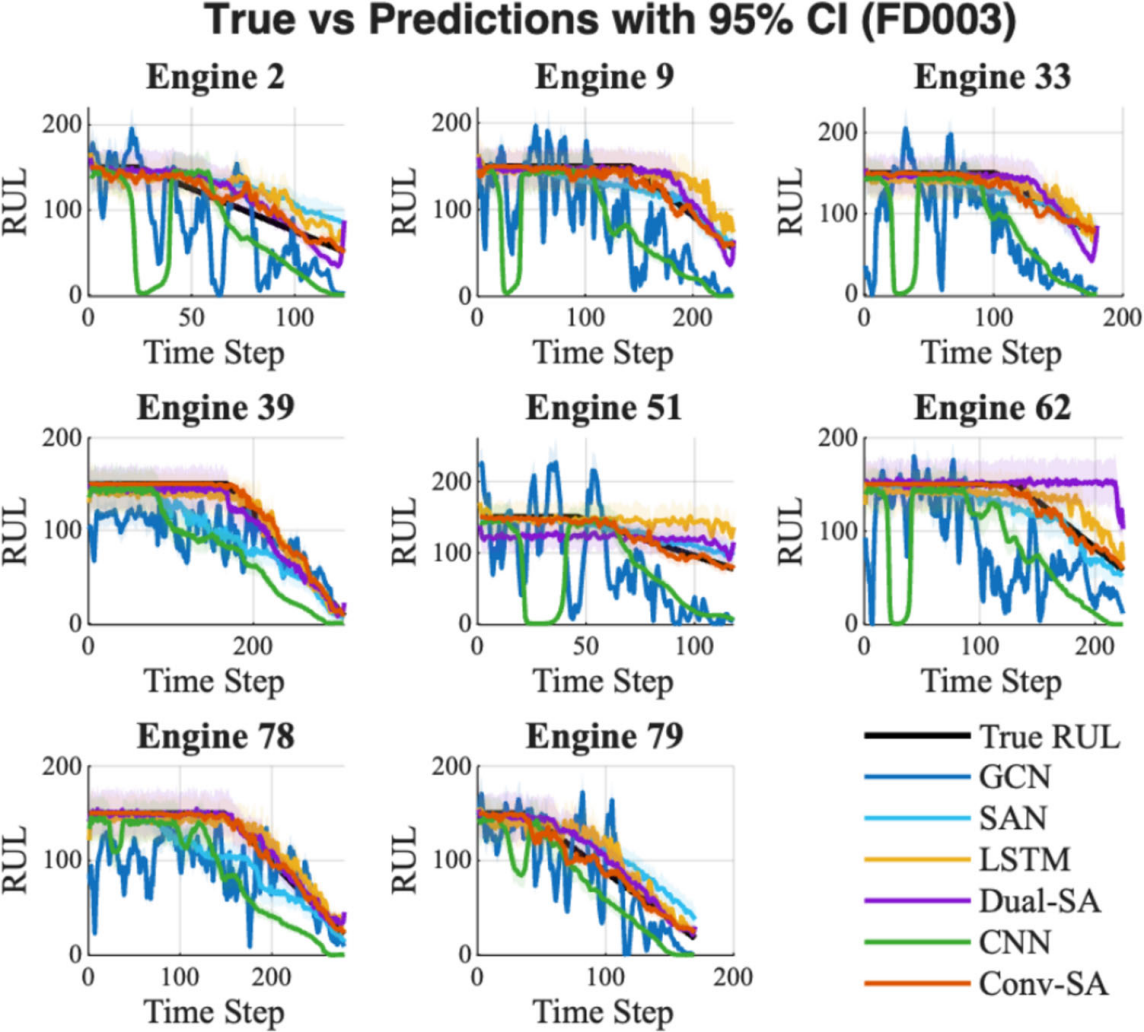
RUL prediction with 95% confidence interval for FD003

Table 4 reports the statistical results of each model for RUL prediction on FD003, while Fig. 6 depicts the lifecycle predictions with the corresponding 95% confidence interval.

**Table 4:** Reliability and superiority evaluation of RUL prediction under FD003

Method name	RMSE $(\overline{x }\pm \sigma )$	Epistemic uncertainty	p-value vs Conv-SA
Conv-SA	$15.72\pm 9.71$	**3.42**	–
GCN	$16.03 \pm 9.78$	10.11	$0.0482$
SAN	$16.19\pm 9.55$	4.72	$0.0546$
LSTM	$18.85\pm 9.73$	10.45	$0.0181$
Dual-SA	$66.21\pm 20.62$	10.11	$5.99\times {10}^{-40}$
CNN	$67.28\pm 21.66$	5.01	$6.73\times {10}^{-41}$

The best performing result for each metric is highlighted in bold

In Table 4, the proposed Conv-SA achieves the lowest RMSE (15.72 ± 9.71), slightly better than GCN (16.03 ± 9.78) and SAN (16.19 ± 9.55), while clearly outperforming the other baselines. Compared with these advanced methods, Conv-SA demonstrates superior overall accuracy and stability. In terms of epistemic uncertainty, Conv-SA again records the lowest value (3.42), indicating higher reliability and confidence in its predictions. By contrast, LSTM and GCN exhibit larger uncertainties (10.45 and 10.11, respectively), suggesting less stable performance. A lower epistemic uncertainty implies that the predicted RUL is more trustworthy, which is especially valuable for practical maintenance decision-making. Furthermore, the p-values confirm that Conv-SA’s improvements over LSTM, Dual-SA, and CNN are statistically significant, whereas the differences with GCN and SAN are relatively small but still highlight Conv-SA’s robustness. In Fig. 6, Conv-SA’s predicted curves (red line) closely follow the ground truth with minimal deviations across most engines. The confidence intervals of Conv-SA are narrower than those of competing models, consistent with the lower epistemic uncertainty observed in Table 4. Conv-SA successfully captures the degradation trend and aligns well with the actual failure progression, while other models such as LSTM, Dual-SA, and CNN often display higher variability, oscillations, or premature convergence, resulting in less reliable lifecycle predictions.

**Fig. 7:**
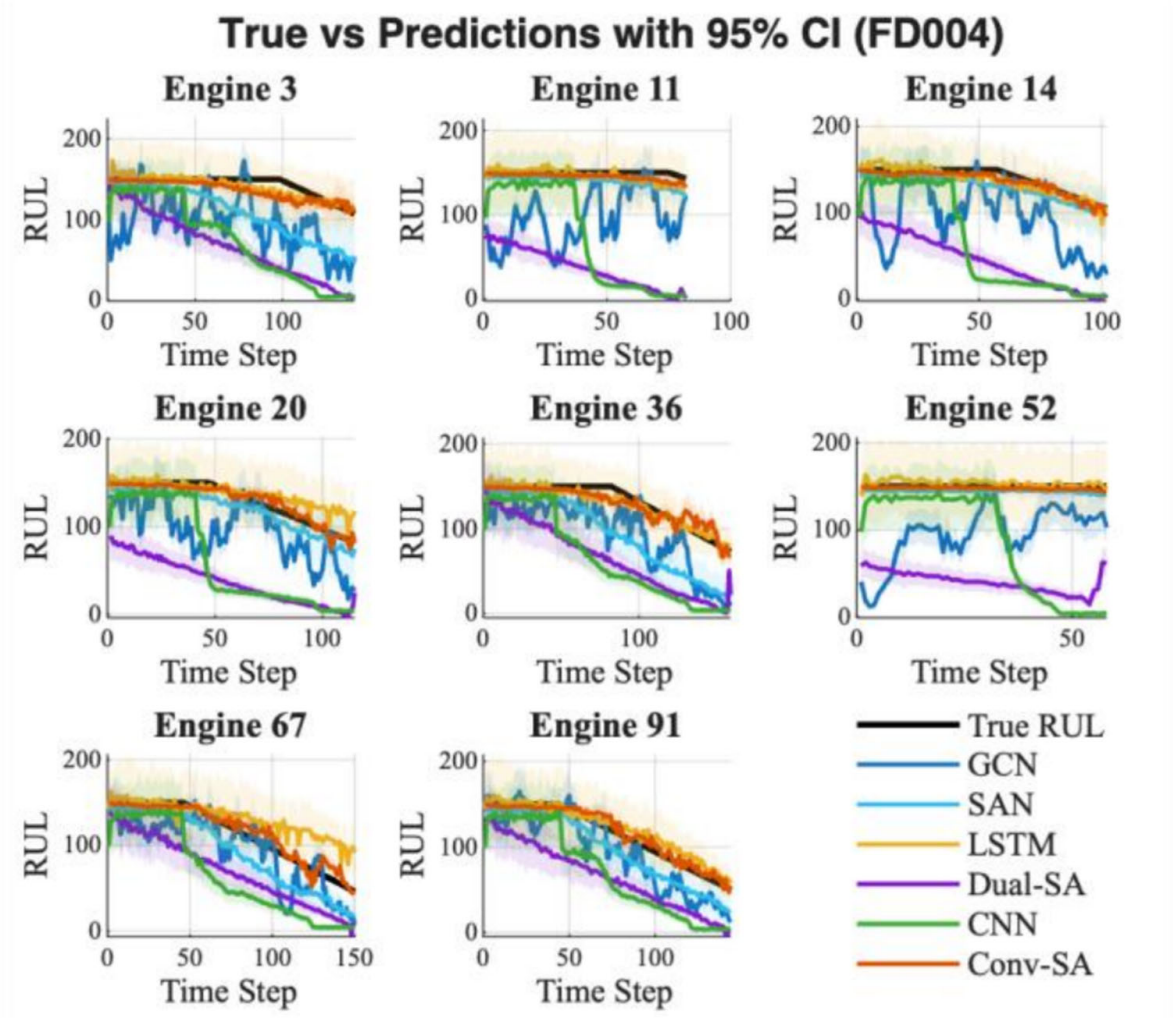
RUL prediction with 95% confidence interval for FD004

Table 5 reports the statistical results of each model for RUL prediction on FD004, while Fig. 7 depicts the lifecycle predictions with the corresponding 95% confidence interval. In Table 4, the proposed Conv-SA achieves the lowest RMSE (21.15 ± 12.42), substantially outperforming all baseline models. Compared with other advanced approaches, Conv-SA demonstrates clear superiority in both prediction accuracy and stability. In terms of epistemic uncertainty, Conv-SA again records the lowest value (4.53), suggesting higher confidence and reliability in its predictions. In contrast, LSTM and CNN show much larger uncertainties (17.90 and 11.20, respectively), indicating less stable performance. Lower epistemic uncertainty implies that the predicted RUL is more trustworthy, which is crucial for effective maintenance decision-making. Moreover, all comparisons yield extremely small p-values, confirming that Conv-SA’s advantage over competing methods is statistically significant. In Fig. 7, Conv-SA’s predicted curves (red line) consistently follow the ground truth with minimal deviations across different engines. The confidence intervals are narrower compared with those of other models, consistent with the uncertainty statistics reported in Table 4. Conv-SA successfully captures the degradation trajectories and aligns well with the actual failure progression. In contrast, competing models such as LSTM, Dual-SA, and CNN often exhibit wider deviations, oscillations, or premature convergence, leading to less accurate lifecycle predictions.

**Table 5:** Reliability and superiority evaluation of RUL prediction under FD004

Method name	RMSE $(\overline{x }\pm \sigma )$	Epistemic uncertainty	p-value vs Conv-SA
Conv-SA	$21.15\pm 12.42$	**4.53**	–
GCN	$45.86\pm 16.16$	9.24	$5.69\times {10}^{-42}$
SAN	$31.58\pm 22.38$	7.31	$3.33\times {10}^{-14}$
LSTM	$26.16\pm 17.92$	17.90	$1.82\times {10}^{-12}$
Dual-SA	$66.66\pm 38.22$	9.55	$1.92\times {10}^{-37}$
CNN	$64.14\pm 25.33$	11.20	$1.28\times {10}^{-55}$

The best performing result for each metric is highlighted in bold

Across all four benchmark datasets, the proposed Conv-SA consistently achieves the lowest RMSE and the smallest epistemic uncertainty, clearly outperforming state-of-the-art approaches in both accuracy and reliability. The statistical tests further confirm that these improvements are highly significant. More importantly, Conv-SA not only captures the degradation trends with minimal deviation from the ground truth but also provides narrower confidence intervals, ensuring stable and trustworthy RUL predictions. Such performance is critical in the prognosis stage of PdM, where reliable RUL estimation directly affects maintenance planning and decision-making. By reducing uncertainty and improving predictive confidence, Conv-SA enhances the safety, robustness, and trustworthiness of AI-based prognosis, thereby promoting the secure and reliable adoption of AI in real-world industrial applications.

### Maintenance optimization

**Fig. 8:**
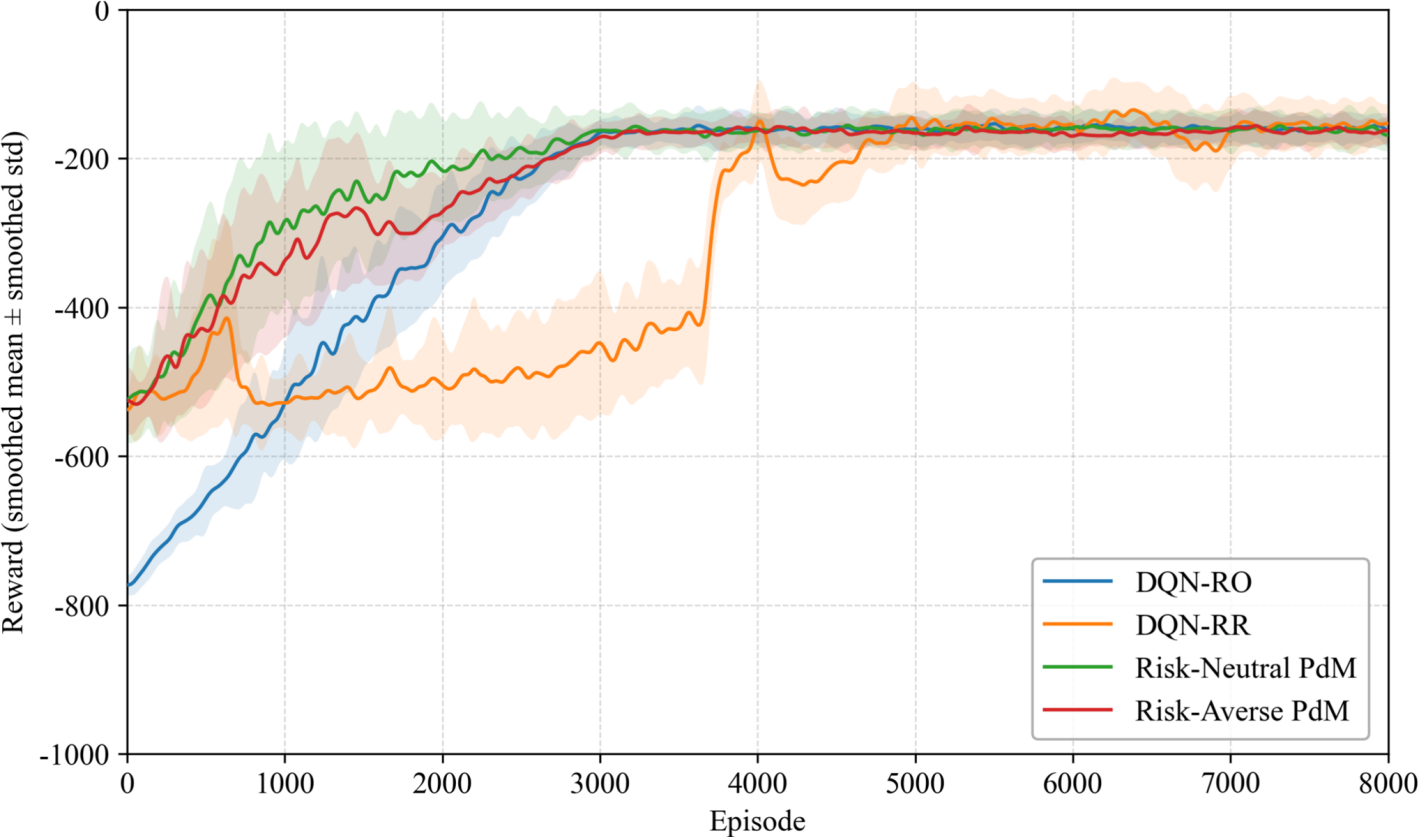
Reward during training for different PdM approach

To validate the effectiveness of the proposed method, we compared it with four benchmark strategies, as shown in Fig. 8. The DQN-RO baseline, which only considers replacements, achieves gradual improvement but is limited by its action space, leading to suboptimal long-term returns. The extended DQN-RR baseline incorporating both repair and replacement exhibits high variance during training and converges slowly, although its asymptotic performance surpasses DQN-RO. The Risk-Neutral PdM, built on QR-DQN without explicit risk sensitivity, converges rapidly to a high-reward policy but exhibits relatively higher fluctuations, reflecting aggressive decision-making. In contrast, the proposed Risk-Averse PdM achieves both high returns and improved stability, converging almost as fast as the Risk-Neutral PdM while maintaining smaller variance. This demonstrates that our method effectively balances cost, risk, and reliability considerations, yielding a more practical and robust predictive maintenance policy.

**Fig. 9:**
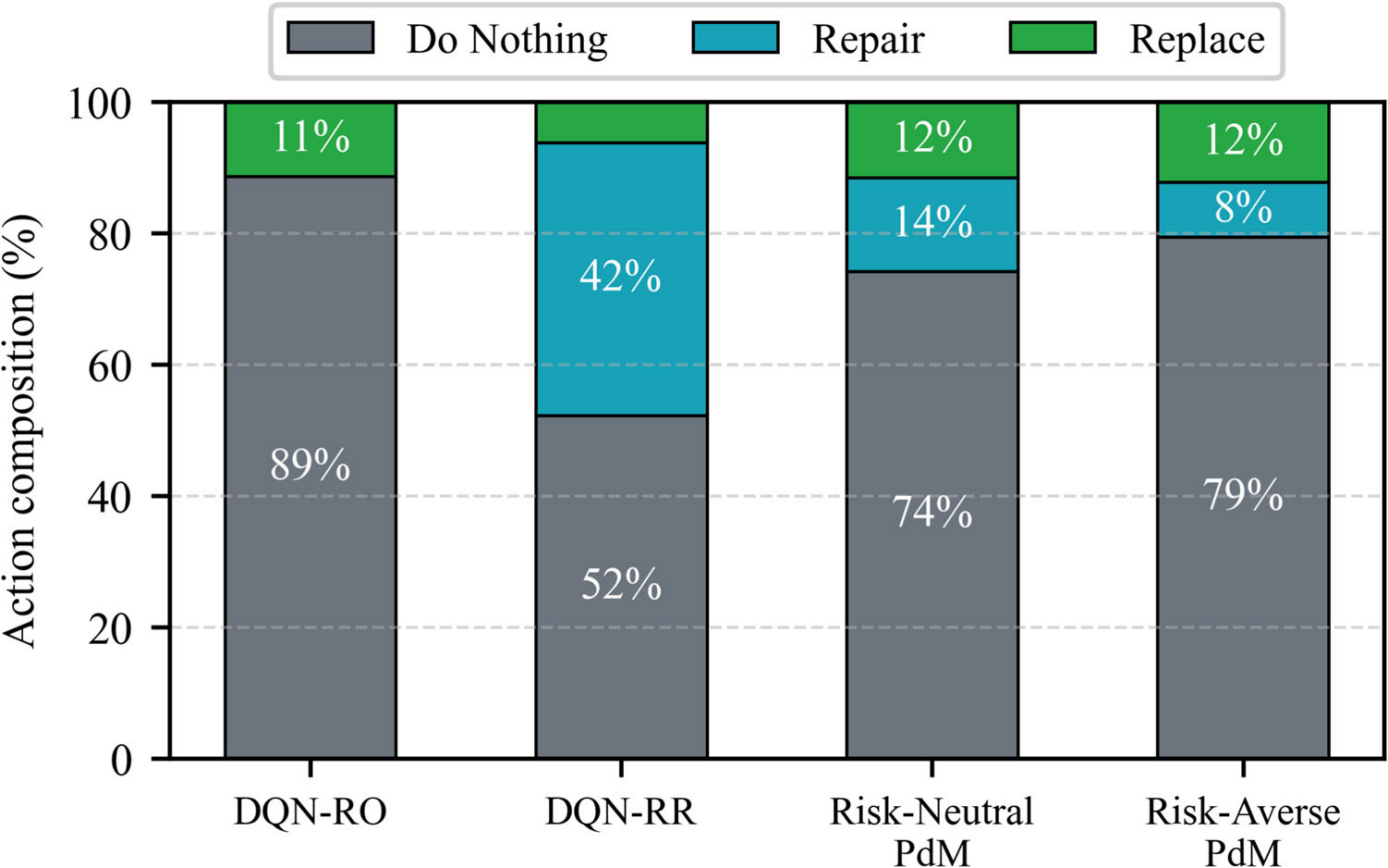
Action composition of different maintenance strategies

Figure 9 illustrates the action composition of the four maintenance strategies. The DQN-RO baseline relies almost exclusively on doing nothing (89%) with occasional replacements (11%), reflecting its limited flexibility. By contrast, the DQN-RR baseline exhibits a much higher proportion of repairs (42%), which reduces costly replacements but results in frequent interventions. The Risk-Neutral PdM strikes a more balanced policy, with 74% do nothing, 14% repairs, and 12% replacements, indicating an aggressive yet diversified decision-making pattern. In comparison, the proposed Risk-Averse PdM adopts a more conservative behavior: it increases the proportion of do nothing (79%) while reducing repairs (8%) and maintaining a similar replacement level (12%). This demonstrates that the proposed method effectively avoids excessive maintenance while ensuring timely replacements, thus achieving a more practical and robust predictive maintenance policy.

**Fig. 10:**
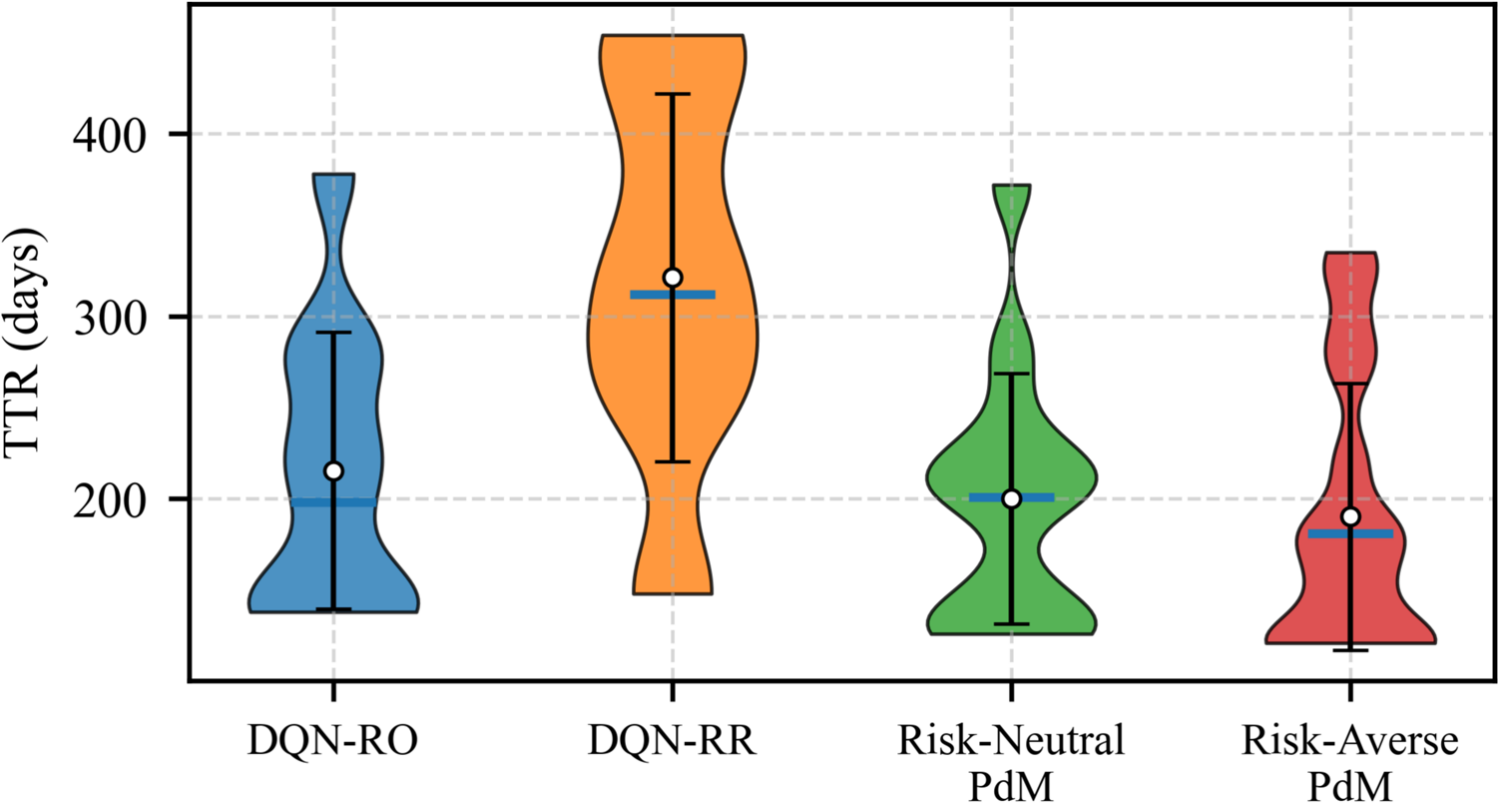
Comparison of repair interval distributions for four maintenance strategies

Figure 10 presents the distribution of TTR under different strategies. The DQN-RO baseline yields moderately long and relatively stable repair intervals, as replacements are the only available actions. The DQN-RR baseline shows the longest median TTR but with the widest spread, indicating highly variable maintenance intervals due to frequent and irregular repair decisions. The Risk-Neutral PdM leads to shorter and more frequent maintenance intervals, with the lowest median TTR but at the cost of higher intervention rates. In contrast, the proposed Risk-Averse PdM achieves a balanced outcome: its median TTR is slightly higher than the Risk-Neutral policy, while the variance is significantly reduced, reflecting a more stable and practical decision-making pattern that avoids both excessive interventions and overly delayed repairs.

**Fig. 11:**
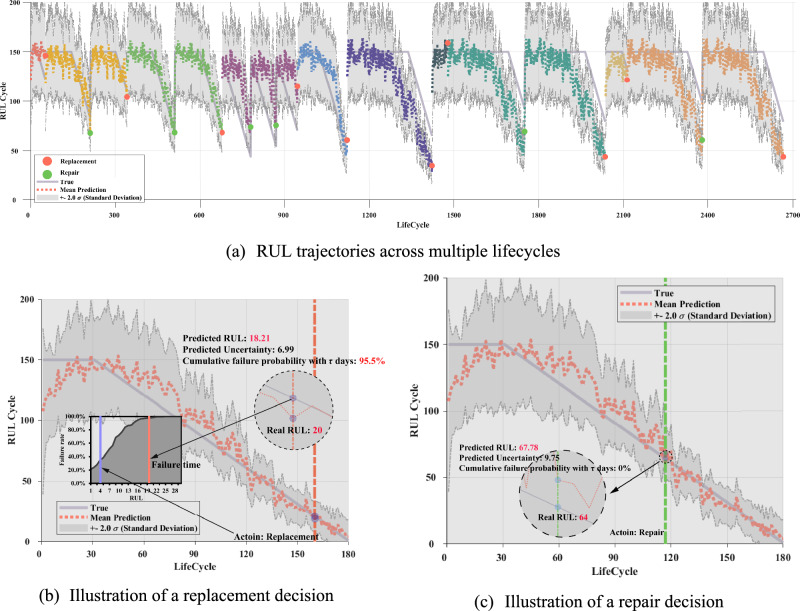
RUL tracking and decision visualization under the proposed PdM framework

Figure 11 illustrates how the proposed PdM framework tracks the RUL and makes maintenance decisions under uncertainty. Panel (a) shows the RUL trajectories across multiple lifecycles, where the predicted mean and confidence bounds closely follow the true RUL, and maintenance actions are triggered before failure. Panel (b) details a replacement case: when the predicted RUL approaches 18.21 with high uncertainty and the failure probability in the next inspection exceeds 95.5%, the agent triggers replacement, which successfully avoids imminent failure (true RUL = 20). Panel (c) highlights a repair case: when the predicted RUL is 67.76 with lower uncertainty and negligible short-term failure risk, the agent opts for repair, partially restoring the lifetime while controlling maintenance cost. These visualizations confirm that the proposed PdM strategy effectively balances safety, cost, and timeliness by adaptively selecting between repair and replacement actions based on predictive RUL and risk assessment.

## Conclusions

This study proposes an AI-driven PdM framework that integrates a powerful RUL prediction model with a distributional reinforcement learning agent based on QR-DQN. The RUL model processes sensor data to provide a more reliable representation of the equipment’s health state, including the expected lifetime, predictive uncertainty, and the probability of failure in the upcoming horizon. Building upon this information, the QR-DQN agent learns the distribution of long-term maintenance returns and makes sequential decisions among multiple actions (do nothing, repair, or replace). By adopting risk-sensitive decision rules, the agent explicitly accounts for uncertainty and failure risk, thereby striking a balance between safety, cost-efficiency, and timeliness of interventions. Experimental results demonstrate that the proposed method outperforms conventional baselines in reducing catastrophic failures, optimizing maintenance schedules, and improving overall reliability for industrial equipment such as CPS and critical infrastructure.

## Data Availability

The datasets generated and/or analyzed during the current study are available from the corresponding author on reasonable request.

## Abbreviations

RUL: Remaining useful life
PHM: Prognostics and health management
CPS: Cyber-physical systems
PdM: Predictive maintenance
O&M: Operation and maintenance
CM: Condition monitoring
DL: Deep learning
CNN: Convolutional neural network
LSTM: Long short-term memory
HSR: Health-state related
MC: Monte Carlo
WTs: Wind turbines
SAN: Self-attention network
BDL: Bayesian deep learning
RL: Reinforcement learning
GA-PSO: Genetic algorithm-particle swarm optimization
Q,K,V: Queries, keys, values in self-attention mechanism
${\mathcal{W}}_{Q}$, ${\mathcal{W}}_{K}$, ${\mathcal{W}}_{V}$: Projection matrices for Q, K, V
Conv-SA: Convolutional networks with self-attention networks
CFP: Cumulative failure probability
DQN: Deep Q-network
QR-DQN: Quantile regression deep Q-network
FFN: Feed-forward network
MLP: Multi-layer perceptron
GCN: Graph convolutional network
Dual-SA: Dual self-attention
